# The Impact of Endothelial Cell Values in Bone Marrow on the Survival of Patients with Multiple Myeloma—A Single-Center Observational Study

**DOI:** 10.3390/jcm14196710

**Published:** 2025-09-23

**Authors:** Krzysztof Gawroński, Nadia Hussein, Elżbieta Rutkowska, Iwona Kwiecień, Agata Raniszewska, Katarzyna Gawrońska, Piotr Rzepecki

**Affiliations:** Department of Hematology, Military Institute of Medicine—National Research Institute, Szaserow Street 128, 04-141 Warsaw, Polandaraniszewska@wim.mil.pl (A.R.); kgawronska@wim.mil.pl (K.G.); przepecki@wim.mil.pl (P.R.)

**Keywords:** multiple myeloma, endothelial cells, survival

## Abstract

**Background:** This study aimed to analyze the survival of patients with multiple myeloma in relation to the value of endothelial cells involved in the process of tumor neoangiogenesis. **Methods:** In this non-randomized observational study, we prospectively evaluated a cohort of 74 adult patients with multiple myeloma who underwent a baseline assessment of the endothelial cell count in their bone marrow and received VCD or VTD anti-myeloma therapy followed by autoPBSCT. They were then evaluated for survival via long-term follow-up. **Results:** The survival of myeloma patients undergoing these therapies was analyzed, and we found that patients with higher endothelial cell counts had higher mortality rates during long-term follow-up. In the group of patients who died, the endothelial cell count was significantly higher (*p* = 0.024). We also observed that patients who initially had >2 osteolytic lesions had higher endothelial cell counts (*p* = 0.021). However, our analysis of endothelial cell count in relation to patient survival using antiangiogenic drugs showed that, in this group, the endothelial cell count was significantly higher in patients who died (*p* = 0.048). **Conclusions:** We found that patients with higher endothelial cell counts and those who did not receive antiangiogenic drugs from the start of therapy had higher mortality rates during long-term follow-up.

## 1. Introduction

Plasma cell myeloma is a cancer originating from B cells in the final stage of differentiation, after recombination of the heavy-chain class of immunoglobulins, which, in such cases, secrete monoclonal protein. We are currently seeing a significant increase in the availability of new drugs, including innovative immunotherapies such as bispecific antibodies, which are an important therapeutic option, especially for patients with relapsed and refractory multiple myeloma. Continuous therapies are increasingly being used, and decisions to start treatment in relapse are made earlier, considering disease activity biomarkers. Optimizing treatment based on evidence from clinical trials is of the utmost importance. Previously, treatment of patients who were not eligible for autologous HSCT was based on protocols using low doses of melphalan, with the addition of newer drugs, i.e., bortezomib, thalidomide, and lenalidomide. Such treatments should be considered as a second option only when it is not possible to initiate therapy based on lenalidomide. The first-line protocols recommended in Poland are DRd (daratumumab, lenalidomide, dexamethasone), DVTd (daratumumab, bortezomib, thalidomide, dexamethasone), RVd (lenalidomide, bortezomib, dexamethasone), or Rd (lenalidomide and dexamethasone). Currently, VCD and VTD regimens with reduced doses of cyclophosphamide, thalidomide, and bortezomib, depending on age and general fitness, are used much less frequently in this group of patients. These regimens were the primary therapies used in 2018, and the patients described in this manuscript were observed based on these regimens.

Another important aspect is the diagnosis of myeloma. In addition to assessing bone-marrow plasma cell counts, their clonality, osteolytic lesions, increasing renal failure parameters, calcium levels, and the presence and levels of monoclonal protein, it is also important to assess cytogenetic risk factors. The current ISS version (R2-ISS) utilizes various available prognostic tools to determine risk based on ISS level (tumor burden), LDH (lactate dehydrogenase) level, and the presence of aberrations identified via FISH testing, such as del(17p), t(4;14), and 1p−/1q+. These features have been combined to define an additive score for stratifying patients with newly diagnosed myeloma to create a uniform prognostic index. Compared to R-ISS, the new R2-ISS adds 1p−/1q+ to the score, and its calculation accounts for the prognostic significance of the coexistence of several cytogenetic abnormalities. The IMGW (2022) recommendations are currently limited to the assessment of t(4;14), t(14;16), del(17p), and 1q/1p status. t(4;14), t(14;16), and del(17p) abnormalities were included in the previous Revised International Staging System (R-ISS, 2015) for risk stratification, which did not include the significance of the 1q/1p abnormality at that time. When we enrolled patients in this study in 2018 and 2019, the assessment of risk stratification factors was not prevalent in Poland, as the modern therapies we have today were not available. At that time, we only focused on whether the patient had renal failure and whether there were any contraindications to high-dose chemotherapy supported by hematopoietic stem-cell transplantation. Additionally, most hematology centers determined the type of therapy based on these aspects. Currently, risk stratification factors must be considered when choosing the type of therapy. Theories about the importance of angiogenesis and endothelial cells in tumor development have been around since the beginning of the 21st century. This later contributed to the use of antiangiogenic drugs in cancer and also in the plasma cell myeloma we analyzed. In our study, we attempted to assess the survival rates of our patients who received therapy with an antiangiogenic drug and those who received therapy without such a drug in the composition [1].

Multiple myeloma (MM) develops mainly in the bone marrow (BM). Extramedullary foci of myeloma are much less common. The bone-marrow microenvironment is therefore an important factor in maintaining the growth, migration, and survival of plasma cells. It appears that the interactions between bone-marrow microenvironment cells, including endothelial cells, and plasma cells play a significant role in disease progression. Bone-marrow neovascularization is a constant feature of MM and goes hand in hand with progression to the leukemic phase [2]. We now know that higher levels of circulating endothelial cells (CECs) are usually associated with poorer patient survival and faster disease progression, particularly in cancers such as glioblastoma multiforme, as they may indicate increased endothelial cell dysfunction or activation [3]. It has also been shown that a decrease in the number of activated CECs during treatment may predict longer progression-free survival in patients with non-small cell lung cancer (NSCLC) [4]. Thalidomide has been confirmed to inhibit angiogenesis by inhibiting the secretion of vascular endothelial growth factor (VEGF), but the exact mechanism by which thalidomide inhibits vascular proliferation during PD is still unclear [5].

However, the relationship between patient survival and endothelial cell values in patients with multiple myeloma has not been sufficiently analyzed to date. Blocking endothelial cell growth with antiangiogenic drugs, i.e., targeting the bone marrow microenvironment, may improve overall survival (OS) in certain cancers, especially when combined with other treatments. It appears that influencing the bone marrow microenvironment, including endothelial cells, with antiangiogenic drugs in multiple myeloma may enhance the effectiveness of anti-myeloma therapy and improve the overall survival of patients. Our work is an attempt to fill this gap in hematology.

## 2. Materials and Methods

We performed an observational, non-randomized study that involved a prospective evaluation of a cohort of 74 adult patients with multiple myeloma and indications for treatment, including the use of the antiangiogenic drug thalidomide in first-line therapy. Patients were treated at the Hematology Clinic of the Military Medical Institute in Warsaw.

This study was conducted in accordance with the Declaration of Helsinki. Blood and marrow samples were taken during routine diagnostics and approved by the Ethics Committee of the Military Institute of Medicine on 10 January 2018 (Military Institute of Medicine Ethics Committee number: KB/234/18). The purpose and procedures of this study were explained to the participants, and their verbal and written informed consent was obtained. It should be emphasized that all patients eligible for inclusion in this study met the criteria to receive standard treatment in 2018–2019, including thalidomide in first-line therapy.

The National Comprehensive Cancer Network developed guidelines for the diagnosis, therapy, monitoring, and supportive care of multiple myeloma (MM). An update was provided in January 2018, detailing guidelines for the therapeutic management of multiple myeloma [1]. NCCN experts classified the drugs used in MM therapy—both for newly diagnosed patients and those with refractory/relapsed myeloma—into three groups: “preferred”, “other recommended”, and “useful in specific circumstances”. There were preferred regimens involving triple-drug therapies based on the use of bortezomib for patients with newly diagnosed multiple myeloma (ND MM) eligible for autologous hematopoietic stem-cell transplantation, as follows: bortezomib/lenalidomide/dexamethasone (VRD) and bortezomib/cyclophosphamide/dexamethasone (VCD). The VCD regimen was the treatment of choice in patients with acute kidney injury, with the possibility of switching to VRD after demonstrating improved kidney function.

However, in 2018–2019, the situation in Poland was different. Firstly, it should be emphasized that first-line treatment with lenalidomide, as recommended by the NCCN, was not reimbursed in Poland. According to the guidelines of the Polish Myeloma Group published in 2017, patients eligible for autoHSCT should have received induction therapy according to the VTD (bortezomib/thalidomide/dexamethasone), VCD (bortezomib/cyclophosphamide/dexamethasone), or PAD (bortezomib/doxorubicin/dexamethasone) protocols.

In this study, we only considered patients who were eligible for treatment with VTD or VCD therapy.

The general eligibility criteria were as follows: (1) aged over 18 years; (2) performance status 0–2 according to the ECOG scale (Eastern Cooperative Oncology Group performance scale); (3) diagnosis of multiple myeloma; (4) presence of indications for treatment in accordance with the guidelines of multiple myeloma: ESMO Clinical Practice Guidelines for diagnosis, treatment, and follow-up [1]; (5) no contraindications to drug use in accordance with the current summary of product characteristics; (6) no hypersensitivity to any of the drugs or excipients of the drugs; (7) patients who were not pregnant or lactating; (8) patients who provided their consent to use contraception in accordance with the current summary of product characteristics; (9) no active and serious infections; and (10) no significant comorbidities or clinical conditions that are contraindications to therapy.

The exclusion criteria were as follows: (1) the occurrence of hypersensitivity symptoms to any of the drugs used or the excipients of the drugs; (2) pregnancy or breastfeeding; and (3) the occurrence of diseases or conditions that, in the opinion of the attending physician, prevent treatment continuation (Table 1).

All patients underwent standard initial examinations to assess their condition, including medical, physical, and biochemical tests and examinations, with a particular focus on kidney function and electrolyte levels (sodium, potassium, and calcium). Imaging tests, especially bone tomography to assess the process of bone osteolysis, and a bone marrow examination were also performed. The patients’ bone marrow was subjected to both a standard assessment to determine the degree of plasma cell infiltration and an additional examination of the endothelial cell activity. The test was performed using flow cytometry. Levels of remission:

- Partial response (PR): A 50% reduction in M-protein.
- Very good partial response (VGPR): A 90% reduction in M-protein.
- Complete response (CR): No detectable M-protein in blood or urine and no visible tumors.
- Non-remission (NR) means failure to meet the PR criterion.

### 2.1. Statistical Methodology

All statistical calculations were performed using the statistical package TIBCO Software Inc. (2017) Statistica (data analysis software system), version 13, https://statistica.software.informer.com/13.3/ (accessed on 12 May 2025).

Quantitative variables were characterized using the arithmetic mean, standard deviation, median, minimum and maximum values (range), and a 95% confidence interval (CI). Qualitative variables were presented using counts and percentages (percentage).

The Shapiro–Wilk test was used to check whether the quantitative variable indicated a normally distributed population. The Levene (Brown–Forsythe) test was used to test the hypothesis of equal variances.

The significance of the differences between the two groups was examined using significance tests: Student’s *t*-test or the Mann–Whitney U-test. The significance of the differences between more than two groups was checked using the F test (ANOVA) or the Kruskal–Wallis test. In the case of there being statistically significant differences between the groups, post hoc tests were used (Tukey’s test for F, Dunn’s test, or the Kruskal–Wallis test).

Correlation analysis was used to calculate Pearson’s and/or Spearman’s correlation coefficients and determine the relationship, strength, and direction between the variables. In all calculations, a significance level of *p* = 0.05 was considered.

### 2.2. Flow Cytometry

**Figure 1:**
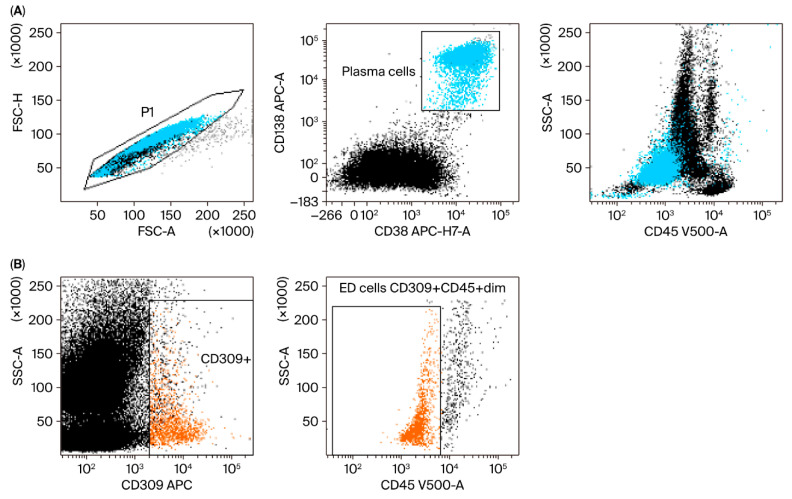
Representative gating strategy in the study group for plasma and endothelial cells (ED cells). (**A**) Plasma cell (blue) gating strategy. FSC-A vs. FSC-H plot: This involves gating cells with equal area and height, thereby removing clumps (cells with a greater FSC-A relative to FSC-H) and debris (cells with very low FSC) (P1 gate). CD138 APC vs. CD38 APC-H7-A plot: Selection of plasma cells based on their expression of antigens. Location of plasma cells on the SSC-A vs. CD45 V500-A plot. (**B**) Endothelial cell (orange) gating strategy: Dot plots of endothelial cells based on their SSC/CD309 APC properties. The next step is to select endothelial cells on the SSC-A/CD45 V500-A plot (phenotypes of cells described in Section 2).

Plasma and endothelial cells were identified when evaluating surface and intracellular antigens via flow cytometry with FACS Canto II BD (Becton Dickinson, Franklin Lakes, NJ, USA). We used an 8-color panel of monoclonal antibodies. The cells were stained with fluorescently labeled antibodies, including CD45-V500, CD19 PE-Cy7, CD138-APC, CD38-APC-H7, CD20-V450, and CD309-APC (BD Biosciences, San Jose, CA, USA), for 20 min at room temperature. After washing, the cells were analyzed within 2 h. For the detection of intracellular markers, including lambda-FITC and kappa-PE (Sigma-Aldrich, St. Louis, MO, USA), an additional step was performed with IntraStain (Dako, Glostrup, Denmark) to fix and permeabilize the membrane. All samples were collected using a standard instrument setup, verified daily using CS&T IVD beads, and acquisition thresholds remained constant throughout the study period with a consistent PMT and compensation configuration. Each sample acquisition included at least 200,000 events to ensure reproducibility. Although this was a single-center study, gating was performed according to a predefined strategy (Figure 1) and independently verified by two experienced cytometrists. Sample identifiers were anonymized during flow cytometry analysis, ensuring operator blindness to clinical outcomes. All analyses were performed at a single center using the same instrument by the same team within a short period of time (2018–2019), which limited sources of inter-study variability. Data were analyzed using DIVA Analysis 8.0.1 software (Becton Dickinson) and Infinicyt 1.8 flow cytometry (Cytognos, Salamanca, Spain). In this representative figure (Figure 1), we show an example patient and the method of labeling the cells studied. Endothelial cells were designated as CD309-positive events and additionally delimited by the CD45-negative antigen. The cutoff point for CD309-positive cells is CD309-negative cells, i.e., the remaining bone-marrow cells.

Plasma cells: CD45+dim CD138+ CD38+.

Endothelial cells: CD45−/+dim CD309+ CD138−. (PMID: 33218322, PMID: 27560136, PMID: 26380236, PMID: 20123847.)

## 3. Results

Seventy-four patients with multiple myeloma were included in this study. All of the included patients were eligible for VTD or VCD treatment. A total of 45 patients (60.81%) underwent VCD therapy, and 29 patients (39.19%) underwent VTD therapy. In all patients, first-line therapy was completed with high-dose chemotherapy using melphalan at a dose of 100–200 mg/m^2^ as monotherapy, supported by autologous hematopoietic cell transplantation (autoPBSCT). In the study cohort, the percentage of women and men was 47.3% and 52.7%, respectively. The average age of patients was 55.5 years (range 48–61 years). Younger patients without serious comorbidities were specifically selected for our observation so that the predicted survival time, excluding hematological disease, would be at least 10 years. All patients were diagnosed with multiple myeloma. The disease stage was assessed according to the Durie–Salmon classification, and 13.1% and 86.9% of cases were classified as in stages 2 and 3, respectively. Patients with osteolytic bone lesions above two and two or fewer foci accounted for 84.1% and 15.9%, respectively.

Our study began in 2018 and focused on patients with confirmed diagnosis and initiation of treatment for multiple myeloma in 2018 and 2019. Subsequently, after completing their treatment according to the above regimens, the patients underwent long-term observation. In addition, our manuscript analyzed the depth of remission achieved by patients. CR was demonstrated in 28 patients, VGPR in 34, PR in 11, and NR in 5 of the analyzed patients. However, it is worth noting that the analyzed patients mainly achieved complete remission (CR) or very good partial remission (VGPR). Only one patient did not achieve remission in our observation, and this patient died shortly after the start of treatment. At the time of writing, among the 74 patients analyzed, 43 died between 2018 and the end of 2024, and 31 are still alive. The average survival time for deceased patients was 3.7 years (range, 0–6 years). Among the deceased patients, 26 (60.47%) received first-line VCD treatment, and 17 (39.53%) received VTD treatment.

It is well-known that patients with myeloma often experience complications in the form of renal failure. Our study also considered kidney function parameters. The mean creatinine value in the study cohort was 1.3 (range: 0.7–4.5), the mean eGFR value was 57 mL/min/m^2^ (range: 18–>90), the average Hb value was 11.8 (range: 9.8–14.1), the average HCT value was 14.8 (range: 0.1–51.1), and the average platelet count was 84.0 (89.8) (range: 33.0–422.0).

In our patients, the endothelial cell count in bone marrow was assessed before the start of therapy. We performed this test using the immunophenotypic method. VTD and VCD therapies were issued for 6 months until autologous hematopoietic stem-cell transplantation (autoPBSCT) was performed. We attempted to separate hematopoietic cells for autotransplantation after a maximum of four cycles of anti-myeloma therapy, as it is known that thalidomide reduces the number of circulating CD34+ stem cells, which hinders the process of collecting cells from peripheral blood. Next, the survival of myeloma patients undergoing the above therapies was analyzed, and it was shown that patients with higher endothelial cell counts had higher mortality rates during their long-term follow-up. The endothelial cell count was significantly higher (*p* = 0.024) and HR 1.12 in the group of patients who died (Table 2).

We also observed that patients who initially had osteolytic lesions > 2 had higher endothelial cell counts (*p* = 0.021). However, our analysis showed that the endothelial cell counts in patients using antiangiogenic drugs who died were also significantly higher (*p* = 0.048).

**Figure 2:**
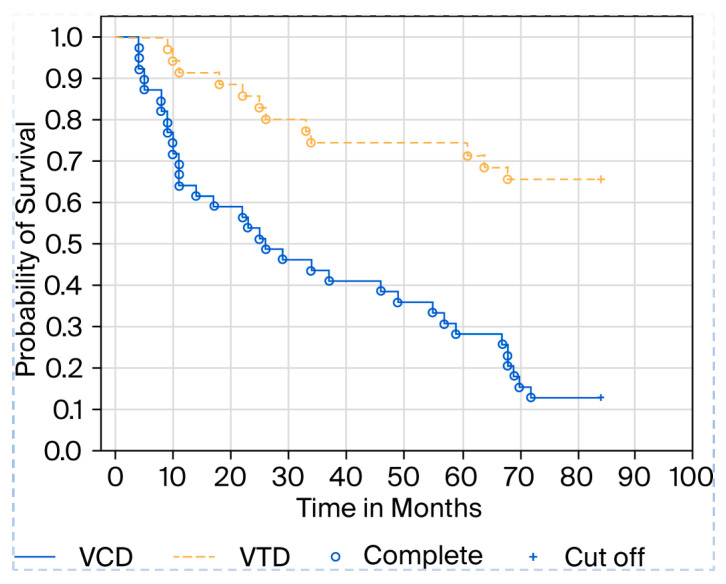
Kaplan–Meier curve showing patient survival depending on the type of therapy.

In our observation, we also conducted a comparative analysis between VTD therapy, which includes the antiangiogenic drug thalidomide, and VCD therapy, which does not include an antiangiogenic drug. As standard, we qualified patients with biochemical indicators of renal failure for VCD. We used a creatinine value of 1.5 mg/dL as the cutoff point for patients with renal failure, with the normal range in our hospital laboratory being up to 1.0 mg/dL. It turns out that survival in the VTD cohort is much higher, averaging 75.7 months, compared to 46 months for VCD, with a statistical significance of *p* = 0.001 (Figure 2).

The most important statistical results are presented in Table 3.

Our study failed to achieve statistical significance in examining the relationship between depth of remission and survival. This may be due to the fact that only 74 patients were analyzed, which is a limitation of our study. After dividing the 74 patients into four groups, the number of analyzed cases became very small (see Table 4).

## 4. Discussion

Understanding the molecular interactions between myeloma and non-hematopoietic cells in the bone marrow is crucial for developing new strategies to improve the effectiveness of myeloma treatment.

Multiple myeloma is a type of blood cancer characterized by the proliferation of malignant plasma cells in the bone marrow. Endothelial cells, in turn, line the inner surface of the blood and lymphatic vessels and play a key role in regulating blood flow, blood pressure, and the formation of new blood vessels. Multiple myeloma is still an incurable disease, so any treatment method that improves prognosis, quality of life, and survival may ultimately represent progress in the treatment of this disease.

### The Relationship Between Myeloma and Endothelial Cells

In the context of myeloma, it has been found that endothelial cells interact with myeloma cells within the bone marrow microenvironment. These interactions may promote the growth and survival of myeloma cells and contribute to the development of drug resistance. Endothelial cells can release various growth factors and cytokines, such as vascular endothelial growth factor (VEGF), which stimulates the proliferation of myeloma cells [6]. In our study, we assessed endothelial cells and considered the effect of the immunomodulatory antiangiogenic drug thalidomide on the survival of patients with myeloma. Thalidomide enhances the anti-tumor immune response but also exhibits cytotoxic activity against multiple myeloma (MM) cells and inhibits tumor-associated angiogenesis [7]. Its newer counterparts have similar effects but are free of many side effects.

The important role that angiogenesis plays in tumor development was initially described in solid tumors. It was not until the 1990s that this role was demonstrated in hematological malignancies, the first model of which was multiple myeloma [8]. Through our study, we confirmed the importance of angiogenesis-inhibiting drugs in the treatment of multiple myeloma, as we demonstrated increased survival in patients with lower baseline endothelial cell counts and improved survival when antiangiogenic drugs were used. Morgan et al. [9] have shown that the continued use of thalidomide significantly improves PFS and may be associated with improved OS. Our observation confirmed this theory. Reyes et al. and Braunstein et al. emphasize the need to investigate the hypotheses that MM and EPC cells originate from multipotent progenitor cells [10], that MM stem cells are capable of self-renewal and differentiation [11], and that they are altered by factors causing identical changes in myeloma cells and EPCs. Research into the differentiation potential of single endothelial cells obtained from MM patients, comparing endothelial cells with cancerous plasma cells at the genetic level, is ongoing. These research findings help us to better understand angiogenesis in myeloma and its significance for the treatment of this disease [12]. Rigolin et al. [13] published data on five patients with MM who had an identical 13q14 deletion in both circulating endothelial cells and bone-marrow plasma cells. In other words, they exhibited the same chromosomal aberration as malignant plasma cells. These results suggest that CECs possibly originate from a common precursor of hemangioblasts, which may give rise to both plasma and endothelial cells, and they also indicate that CECs derived from MM have a direct influence on tumor angiogenesis and possibly on disease spread and progression. In their research, Yu M et al. demonstrated that angiogenesis was closely correlated with the prognosis of MM patients. Immunohistochemistry confirmed that high microvessel density could indicate poor prognosis. Assessing the signature of AAGs (angiogenesis-associated genes) facilitated the prediction of patient response to immunotherapy and the targeting of more effective immunotherapy strategies [14]. Multiple myeloma remains an incurable disease that poses significant therapeutic challenges. Minnie et al. noted in their research that a breakthrough in myeloma treatment may occur thanks to the emergence of many new immunotherapies [15]. We are currently seeing a significant increase in the availability of new drugs, including innovative immunotherapies such as bispecific antibodies, which are an important therapeutic option, especially for patients with relapsed and refractory multiple myeloma. Continuous therapies are being used more and more often, and decisions to start treatment for relapse are being made earlier, taking into account biomarkers of disease activity. Great importance is attached to optimizing treatment based on evidence from clinical trials. In addition to new treatments for myeloma, efforts are underway to identify risk factors. The current revision of the ISS (R2-ISS) uses various available prognostic tools to determine risk based on the Revised Multiple Myeloma International Staging System (R-ISS) (tumor burden), LDH (lactate dehydrogenase) level, and the presence of aberrations identified in FISH testing, such as del(17p), t(4;14), and 1p−/1q+ [16].

Since the early 21st century, it has been established that the progression of multiple myeloma is accompanied by increased bone-marrow angiogenesis, but there is still controversy as to whether angiogenesis is a side effect or a driving force behind the progression of multiple myeloma [17]. Therefore, we attempted to assess whether one of the elements of angiogenesis, namely, endothelial cells, and the use of antiangiogenic drugs could affect the survival of patients with multiple myeloma. We can say that our observation was partially successful, as it can be seen that patients with initially higher endothelial cell counts who were treated with a regimen containing thalidomide had higher survival rates.

Combining antiangiogenic therapies with immune checkpoint inhibitors has become an attractive strategy, although there is still a long way to go, considering the difficulties in controlling the immune system, toxicity, and side effects, among other factors [17,18]. We have also drawn conclusions about the importance of antiangiogenic medicines. A limitation of our study is that we did not evaluate endothelial progenitor cells (EPCs, CD34^+^CD133^+^KDR^+^) [19,20] or VEGFR2-positive hematopoietic cells in inflammatory settings [21,22], which could have been included in our gating region. Rare progenitor populations or leukocytes could have influenced the measured endothelial cell pool. This potential overlap in results could lead to an overestimation of endothelial cell numbers, particularly in patients with systemic inflammation. Nevertheless, the consistency of our results across the cohort and their concordance with the known biological role of angiogenesis in multiple myeloma support the validity of our findings. Future studies should utilize broader multiparametric flow cytometry to precisely separate mature endothelial cells from subsets of endothelial progenitors and inflammatory VEGFR2-positive hematopoietic cells.

Nowadays, there has been tremendous progress in the treatment of cancer, including myeloma. Recently, bispecific antibodies have been introduced into treatment, which, on the one hand, bind and activate T lymphocytes, and, on the other hand, bind to plasma cell receptors. Redirecting T cells to specifically target and kill cancer cells has been confirmed as an effective anti-cancer strategy in clinical practice. However, the immunosuppressive nature of the tumor microenvironment can be a major obstacle to T cell therapy. For example, the bone marrow (BM) niche is considered an immunologically privileged site in a state of equilibrium, allowing for normal hematopoiesis and immune cell production. Furthermore, in hematologic malignancies, the BM niche protects tumor stem cells, minimizing the efficacy of several anti-cancer drugs, including chemotherapy, targeted small molecule inhibitors, and antibody-based therapies [23,24]. The demonstration in our studies that increased endothelial cell values shorten the survival of patients with myeloma, while the use of the immunomodulatory drug thalidomide prolongs survival, may lead to the hypothesis that bispecific antibodies should be used in combination with drugs that affect the bone-marrow microenvironment, which will further increase their effectiveness in the treatment of refractory forms of multiple myeloma.

## 5. Conclusions

Our observations indirectly suggest that patients with higher endothelial cell counts have a higher mortality rate. Patients who did not receive the antiangiogenic drug thalidomide from the start of therapy also had a higher mortality rate during long-term follow-up, as shown in the attached Kaplan–Meier curve. In everyday practice, the need to use antiangiogenic drugs as part of myeloma therapy should be considered. The drug currently used in first-line therapy is lenalidomide, a derivative of thalidomide. It also appears that in everyday practice, the number of endothelial cells may be one of many prognostic factors. It seems that patients with high endothelial cell counts should be treated with therapies that also include antiangiogenic drugs, e.g., DVTd and DRd, as is the current trend. In our observation, thalidomide appears to prolong overall survival in patients with myeloma. Since thalidomide affects neoangiogenesis, i.e., endothelial cells, it can be assumed that drugs such as thalidomide and its newer successors must be considered in modern myeloma therapies. Our results suggest that the number of endothelial cells in the bone marrow may be an independent prognostic factor for survival in patients with multiple myeloma. A shortcoming of our observation is that we should also evaluate endothelial cells in the period after therapy and, possibly, during progression; however, we were unable to obtain consent from the bioethics committee for such a procedure. We received permission to conduct research only on patients who had blood and bone marrow samples taken as part of routine diagnostics prior to standard therapy, and our study was treated as an additional study. However, a longer observation period and multicenter studies are necessary to confirm our findings.

## Figures and Tables

**Table 1:** Basic characteristics of the study group in terms of gender, age, ECOG performance status, and clinical stage of disease according to the Durie–Salmon classification.

**Cohort**	74
**Sex**	
Female	47.3%
Male	52.7%
**Age**	
Mean	55.5
Range	48–61
**Diagnosis**	
Myeloma multiplex	100%
**ECOG**	
0	2 (2.7%)
1	42 (56.76%)
2	30 (40.54%)
**Durie–Salmon classification**	
I	0
II	9 (12.14%)
III	65 (87.86%)
**VTD Therapy**	29 patients
Endothelial cell mean	3.43/µL
Hemoglobin mean	11.1 g/dL
Osteolysis	22 patients
Creatinine mean	1.1 mg/dL
**VCD Therapy**	45 patients
Endothelial cell mean	4.93/µL
Hemoglobin mean	10.9 g/dL
Osteolysis	38 patients
Creatinine mean	1.7 mg/dL
**Type of remission**	
CR	28
VGPR	34
PR	11
NR	1

CR—complete remission, VGPR—very good partial remission, PR—partial remission, NR—no remission.

**Table 2:** An analysis of the relationship between endothelial cell values and mortality.

	Death (*n* = 43)	Survival (*n* = 16)	*p*-Value ^1^	Hazard Ratio (HR)
Endothelial cells/µL			0.024	1.12
(SD)	1.544	0.69		
median (IQR)	3.77	1.71		

^1^ Mann–Whitney U-test.

**Table 3:** An analysis of the relationship between endothelial cell values and survival in patients with osteolytic lesions > 2.

	Death (*n* = 25)	Survival (*n* = 35)	*p*-Value ^1^	Hazard Ratio (HR)
Endothelial cells			0.048	1.29
(SD)	2.743	1.67		
median (IQR)	4.65	1.75		

^1^ Mann–Whitney U-test.

**Table 4:** The most important statistical results.

		SD	Level *p*	Hazard Ratio (HR)	95% PU HR Low	95% PU HR Up
Creatinine [mg/dL]		0.14	0.02	0.98	0.91	1.05
Hb [g/dL]		0.07	0.04	0.87	0.76	1.00
Osteolysis (+)		0.34	0.27	1.46	0.75	2.85
Endothelial cells/µL		0.21	0.03	1.88	0.95	7.72
Kind of therapy	VCD	0.35	0.00	3.14	2.10	8.15
	VTD	0.25	0.01	4.16	2.01	7.03
	CR	0.15	0.32	1.24	1.11	2.25
Kind of remission	VGPR	0.19	0.23	1.36	1.01	2.03
	PR	0.33	0.22	1.77	1.12	2.01
	NR	-	-	-	-	-

## Data Availability

The original contributions presented in this study are included in the article. Further inquiries can be directed to the corresponding author.
